# No convincing association between genetic markers and respiratory symptoms: results of a GWA study

**DOI:** 10.1186/s12931-016-0495-4

**Published:** 2017-01-10

**Authors:** Xiang Zeng, Judith M. Vonk, Kim de Jong, Xijin Xu, Xia Huo, H. Marike Boezen

**Affiliations:** 1 Department of Epidemiology, University of Groningen, University Medical Center Groningen, 1 Hanzeplein, Groningen, 9700RB The Netherlands; 2 Groningen Research Institute for Asthma and COPD (GRIAC), University of Groningen, University Medical Center Groningen, 1 Hanzeplein, Groningen, 9700RB The Netherlands; 3 Laboratory of Environmental Medicine and Developmental Toxicology, and Provincial Key Laboratory of Infectious Diseases and Molecular Immunopathology, Shantou University Medical College, 22 Xinling Road, Shantou, 515041 China; 4 School of Environment, Guangdong Key Laboratory of Environmental Pollution and Health, Guangzhou Key Laboratory of Environmental Exposure and Health, Jinan University, Guangzhou, 510632 China

**Keywords:** GWAS, Genetic, Respiratory symptoms, General population, cohorts

## Abstract

**Background:**

Respiratory symptoms are associated with accelerated lung function decline, and increased hospitalization and mortality rates in the general population. Although several environmental risk factors for respiratory symptoms are known, knowledge on genetic risk factors is lacking. We aim to identify genetic variants associated with respiratory symptoms by genome-wide association (GWA) analyses.

**Methods:**

We conducted the first GWA study on cough, dyspnea and phlegm among 7,976 participants in the LifeLines I cohort and used the LifeLines II cohort (*n =* 5,260) and the Vlagtwedde-Vlaardingen cohort (*n =* 1,529) for replication.

**Results:**

We identified 50 SNPs that were assessed for replication. Rs16918212, located in the alpha-2-macroglobulin pseudogene 1 (*A2MP1*), was associated with cough in both the identification (odds ratio (OR) = 0.72, *p =* 5.41 × 10^−5^) and the meta-analyzed replication cohorts (OR = 0.83, *p =* 0.033). No other significant replicated associations were found.

**Conclusions:**

Given that only 1 out of 50 SNPs showed significant replication (i.e. 2%) we conclude that we did not find a convincing association between genetic markers and respiratory symptoms. Since, environmental exposures are important risk factors for respiratory symptoms, the next step is to perform a genome-wide interaction (GWI) study to identify genetic susceptibility loci for respiratory symptoms in interaction with known harmful environmental exposures.

**Electronic supplementary material:**

The online version of this article (doi:10.1186/s12931-016-0495-4) contains supplementary material, which is available to authorized users.

## Background

The presence of respiratory symptoms, such as chronic cough, dyspnea and phlegm, is associated with lower lung function [1, 2] and with mortality due to several causes of death [3–5]. Respiratory symptoms have been regarded as important markers of accelerated lung function decline [6, 7] and development of asthma [8].

It is known that cigarette smoking [9], allergy [10, 11], air pollution [12, 13] and occupational exposures [14, 15] are risk factors for respiratory symptoms. However, not all exposed subjects develop respiratory symptoms, which suggests that a genetic component may be involved in the development of respiratory symptoms. Previous studies reported associations between respiratory symptoms and specific genetic loci using candidate gene studies [16, 17]. To date, only one genome-wide association (GWA) study has investigated genetic susceptibility of respiratory symptoms (i.e. Chronic mucus hyper-secretion) [18]. Genetic susceptibility to develop respiratory symptoms such as cough, dyspnea, and phlegm has not been studied up until now using GWA methods.

In the current study, we conducted several GWA analyses, i.e. on cough, dyspnea and phlegm, in 7,976 Caucasians of Dutch descent from the large population-based LifeLines I cohort study to identify common genetic variants associated with respiratory symptoms. We used the LifeLines II cohort and the Vlagtwedde-Vlaardingen cohort to replicate our initial findings.

## Methods

### Identification cohort

Genotyped individuals from the first data release of the LifeLines cohort study (2006–2011, LifeLines I) with full data on all covariates were included (*n =* 7,976). The LifeLines cohort study is a prospective population-based cohort studying health and health-related behavior of subjects from the three Northern provinces of the Netherlands [19, 20].

### Replication cohorts

We included 5,260 subjects from the second data release from the LifeLines cohort study (2006–2011, LifeLines II) and 1,529 subjects from the last survey (1989/1990) from the Vlagtwedde-Vlaardingen cohort [21, 22], a prospective general population based cohort including Caucasians of Dutch descent, to replicate our initial findings.

### Ethics, consent and permissions

Participants provided written informed consent. The study was approved by the Medical Ethics Committee of the University Medical Center Groningen, Groningen, The Netherlands (ref. METc 2007/152).

### Genotyping and quality control

Genome-wide genotyping was performed in the identification and replication cohorts using IlluminaCytoSNP-12 arrays. The IlluminaCytoSNP-12 is an oligonucleotide chip designed to have a uniform spacing of markers across all chromosomes, with the majority of the markers on this chip reflecting common SNPs: 93% of the 301,232 markers on this chip reflect bi-allelic SNP markers. The applied genotyping quality control criteria in the LifeLines cohort and the Vlagtwedde-Vlaardingen cohort have been described before [19, 20]: Samples with call-rates of less than 95% were excluded as were samples of non-Caucasians and first degree relatives. SNPs were excluded if they had a genotype call-rate < 95%, minor allele frequency (MAF) < 1%, or a Hardy-Weinberg equilibrium (HWE) *p-*value < 10^−4^. In the LifeLines cohort 227,981 SNPs were included and in the Vlagtwedde-Vlaardingen cohort 242,926 SNPs were included.

### Respiratory symptoms

Cough, dyspnea, and phlegm were defined by standardized questionnaires from the European Community Respiratory Health Survey (ECRHS) [23]. Cough was defined as at least one positive answer to the questions: “do you usually cough first thing in the morning in the winter?” or “do you usually cough during the day, or at night, in winter?”. Dyspnea was defined as a positive answer to the question: “are you troubled by shortness of breath when hurrying on level ground or walking up a slight hill or stairs at normal pace?”. Phlegm was defined as at least one positive answer to the questions: “do you usually bring up any phlegm from your chest first thing in the morning in winter?” or “do you usually bring up any phlegm from your chest during the day, or at night, in winter?”.

### Statistical analysis

The data are presented as median (min-max) for continuous variables and as frequencies (percentages) for categorical variables. The GWA analyses on the presence of the respiratory symptoms cough, dyspnea, and phlegm were performed using PLINK version 1.07 [24]. We used an additive genetic model adjusted for age, sex, and current smoking. SNPs with a *p-*value < 10^−4^ in the identification analysis were taken forward for replication. Replication analysis was performed by analyzing the two replication cohorts separately using logistic regression model in PLINK version 1.07 [24] and subsequently meta-analyzing effect estimates from both cohorts. Significant replication was defined as a fixed effect meta-analysis *p-*value < 0.05 and an effect estimate in the same direction as in the identification GWA study. SNP annotation was performed using HaploReg version 4 (Broad Institute).

## Results

Demographic characteristics and the prevalence of respiratory symptoms in the study cohorts are summarized in Table 1. In the identification cohort LifeLines I, the median age of subjects was 47 years old, 43% were male, and 24% were current smokers. The replication cohorts were comparable with the identification cohort with respect to demographic characteristics. The prevalence of respiratory symptoms in the LifeLines cohorts and Vlagtwedde-Vlaardingen cohort varied from 10 to 22%.

**Table 1:** Characteristics of the subjects included in the identification (LifeLines I) and replication (LifeLines II and Vlagtwedde-Vlaardingen) cohorts

	Identification LifeLines I	Replication	
		LifeLines II	Vlagtwedde-Vlaardingen
N	7,976	5,260	1,521
Male, *n* (%)	3,420 (43)	2,112 (40)	805 (53)
Age (yrs), median (min-max)	47 (18–88)	48 (20–89)	53 (35–91)
Current smokers, *n* (%)	1,904 (24)	1,051 (20)	548 (36)
Smoking status, *n* (%)			
Ever	4,737 (60)	3,050 (59)	1,054 (69)
Never	3,208 (40)	2,133 (41)	467 (31)
Respiratory symptoms, *n* (%)			
Cough	1275 (16)	776 (15)	175 (12)
Dyspnea	1097 (17)	749 (17)	338 (22)
Phlegm	872 (11)	534 (11)	147 (10)

The Manhattan plots of the GWAS of cough, dyspnea and phlegm are shown in Additional file 1: Figures S1, S2 and S3 respectively. A total of 17 SNPs, 19 SNPs and 14 SNPs were identified for cough (Table 2), dyspnea (Table 3) and phlegm (Table 4) in the identification analyses in LifeLines I, respectively, and taken forward for replication in LifeLines II and Vlagtwedde-Vlaardingen. Rs16918212 (OR = 0.72, *p =* 5.41 × 10^−5^ in identification; OR = 0.83, *p =* 0.033 in replication), located on *A2MP1*, was significantly associated with cough in the replication cohorts with the same direction of effect as in the identification cohort (Table 2). The replication analyses on dyspnea and phlegm showed no significant replication (Table 3 and Table 4).

**Table 2:** Top SNPs (*n =* 17) associated with cough in the GWA study (all *P <* 1.0 × 10^−4^)

SNPs	Chr	Gene	A1	MAF	Identification LifeLines I		Replication				Meta-analysis Replication	
							LifeLines II		Vlagtwedde-Vlaardingen			
					OR	P	OR	P	OR	P	OR	P
rs11813494	10	638 kb 3′ of SFTA1P	G	0.24	1.279	1.12E-06	1.110	0.114	0.776	0.096	1.049	0.427
rs840952	2	60 kb 5′ of SPRED2	G	0.10	1.212	1.09E-05	1.168	0.006	0.814	0.169	1.035	0.401
rs7633390	3	27 kb 5′ of ZNF717	T	0.18	1.237	2.24E-05	1.037	0.581	0.865	0.305	1.005	0.938
rs4977230	9	SLC24A2	T	0.71	0.817	3.35E-05	1.020	0.744	0.986	0.910	1.013	0.806
rs4780334	16	CIITA	G	0.69	1.209	3.78E-05	0.985	0.802	0.875	0.295	0.964	0.500
rs844033	15	126 kb 5′ of NDN	C	0.91	0.702	3.84E-05	1.163	0.115	1.213	0.342	1.172	0.068
rs422564	5	3.9 kb 3′ of TBCA	T	0.21	1.201	3.97E-05	1.035	0.549	1.196	0.123	1.065	0.222
rs12153520	5	77 kb 3′ of HINT1	A	0.16	1.259	4.48E-05	1.012	0.875	1.020	0.904	1.013	0.844
rs893966	1	EPHB2	T	0.50	1.248	4.72E-05	0.992	0.909	0.909	0.532	0.975	0.707
rs6775462	3	ROBO2	C	0.50	0.739	4.81E-05	0.952	0.588	1.124	0.497	0.987	0.869
rs4766584	12	ACACB	G	0.45	0.821	5.15E-05	0.929	0.234	0.994	0.964	0.941	0.274
rs16918212	12	A2MP1	A	0.20	0.717	5.41E-05	0.882	0.196	0.613	0.021	0.828	0.033
rs2623166	8	GEM	T	0.13	1.293	7.68E-05	0.994	0.948	0.635	0.029	0.928	0.360
rs17729233	18	DSG4	G	0.44	1.257	7.89E-05	1.034	0.668	0.997	0.982	1.026	0.714
rs2850106	21	HLCS	G	0.12	0.839	8.29E-05	1.050	0.384	0.998	0.989	1.040	0.434
rs10758982	9	PTPRD	G	0.34	1.211	8.36E-05	0.967	0.602	0.827	0.163	0.939	0.284
rs2845804	21	HLCS	C	0.18	0.841	9.93E-05	0.976	0.663	1.090	0.461	0.996	0.941

**Table 3:** Top SNPs (*n =* 19) associated with dyspnea in the GWA study (all *P <* 1.0 × 10^−4^)

SNPs	Chr	Gene	A1	MAF	Identification LifeLines I		Replication				Meta-analysis Replication	
							LifeLines II		Vlagtwedde-Vlaardingen			
					OR	P	OR	P	OR	P	OR	P
rs10754237	1	LHX9	T	0.03	1.883	1.80E-06	0.921	0.642	1.523	0.057	1.120	0.412
rs658121	18	LAMA1	T	0.14	1.349	2.70E-06	1.039	0.638	0.776	0.070	0.965	0.614
rs6428425	1	8.1 kb 3′ of LHX9	G	0.97	1.864	2.90E-06	0.912	0.602	1.528	0.056	1.067	0.395
rs13388308	2	ATF2	T	0.19	0.751	4.59E-06	0.997	0.961	0.939	0.581	0.980	0.739
rs7236872	18	208 kb 3′ of CBLN2	A	0.53	1.237	7.79E-06	1.033	0.583	0.956	0.626	1.010	0.834
rs2243335	21	324 kb 5′ of MIR802	C	0.30	0.790	9.91E-06	1.014	0.821	1.077	0.438	1.032	0.540
rs4599120	2	4.3 kb 3′ of LOC84931	A	0.08	1.390	2.11E-05	0.942	0.551	0.895	0.466	0.927	0.368
rs12698902	7	AUTS2	G	0.27	1.239	3.73E-05	1.052	0.425	1.141	0.192	1.077	0.170
rs6977656	7	AUTS2	T	0.19	1.267	4.05E-05	1.065	0.375	1.109	0.358	1.077	0.214
rs2898237	21	329 kb 5′ of MIR802	G	0.30	0.805	4.21E-05	1.025	0.689	1.120	0.228	1.053	0.318
rs10231884	7	LOC100505881	A	0.58	0.817	4.61E-05	0.998	0.972	0.961	0.666	0.987	0.793
rs13232100	7	AUTS2	G	0.40	1.217	4.92E-05	1.036	0.544	1.130	0.197	1.062	0.233
rs11154774	6	22 kb 3′ of LOC154092	G	0.40	1.215	5.25E-05	1.083	0.173	1.083	0.173	1.081	0.113
rs7642234	3	DNAH12	A	0.26	1.253	6.30E-05	0.943	0.402	1.047	0.675	0.972	0.653
rs836692	2	30 kb 5′ of B3GALT1	T	0.32	1.225	6.44E-05	1.048	0.453	1.074	0.456	1.056	0.303
rs6852190	4	LIMCH1	T	0.36	0.813	6.88E-05	1.202	0.002	0.981	0.845	1.135	0.014
rs2063621	3	CADPS	G	0.78	0.797	6.94E-05	1.108	0.125	1.142	0.212	1.118	0.049
rs10180670	2	225 kb 5′ of SOX11	A	0.17	0.775	7.00E-05	0.987	0.859	0.956	0.682	0.978	0.708
rs1391412	16	294 kb 3′ of TERF2IP	A	0.44	0.826	8.37E-05	1.047	0.429	1.010	0.915	1.036	0.473

**Table 4:** Top SNPs (*n =* 14) associated with phlegm in the GWA study (all *P <* 1.0 × 10^−4^)

SNPs	Chr	Gene	A1	MAF	Identification LifeLines I		Replication				Meta-analysis Replication	
							LifeLines II		Vlagtwedde-Vlaardingen			
					OR	P	OR	P	OR	P	OR	P
rs4818199	21	79 kb 3′ of NCRNA00323	T	0.40	0.781	3.28E-06	0.936	0.314	1.048	0.711	0.959	0.470
rs13285576	9	488 kb 3′ of CYLC2	A	0.16	0.724	1.68E-05	1.022	0.800	0.847	0.324	0.986	0.827
rs6828886	4	171 kb 3′ of PAPSS1	C	0.42	0.794	1.81E-05	0.982	0.780	1.035	0.789	0.993	0.904
rs6040994	20	30 kb 3′ of BTBD3	T	0.15	1.371	1.93E-05	0.930	0.481	1.055	0.794	0.954	0.607
rs9643995	8	SGCZ	T	0.03	0.471	2.79E-05	1.402	0.028	0.987	0.971	1.331	0.049
rs9831020	3	107 kb 3′ of LOC100507098	G	0.13	0.691	4.44E-05	1.014	0.892	0.988	0.948	1.008	0.933
rs7680755	4	230 kb 3′ of PAPSS1	T	0.41	0.801	4.66E-05	0.982	0.789	1.030	0.815	0.992	0.896
rs17534243	1	24 kb 3′ of LOC339442	G	0.21	1.265	4.91E-05	0.954	0.546	0.911	0.538	0.945	0.412
rs1156855	15	PAQR5	A	0.39	0.809	5.25E-05	1.026	0.700	1.221	0.126	1.063	0.300
rs6075458	20	145 kb 5′ of SLC24A3	A	0.20	0.759	5.79E-05	1.035	0.676	1.051	0.742	1.039	0.598
rs17792478	6	SENP6	G	0.33	1.233	5.92E-05	1.086	0.219	0.896	0.390	1.042	0.488
rs8025182	15	PAQR5	T	0.39	0.812	6.79E-05	1.016	0.812	1.269	0.063	1.064	0.289
rs6786757	3	SLC9A9	G	0.09	1.411	8.57E-05	1.136	0.290	1.092	0.714	1.127	0.266
rs7633390	3	27 kb 5′ of ZNF717	T	0.20	1.255	9.25E-05	0.970	0.691	0.994	0.967	0.975	0.710

In addition, we performed GWA analyses on chronic cough and phlegm (both defined as cough or phlegm for at least 3 months per year) and found no significant replication in these analyses either (Additional file 1: Tables S1 and S2).

## Discussion

To the best of our knowledge, this is the first GWA study assessing genetic variants associated with cough, dyspnea, and phlegm. In the identification cohort, we identified 17, 19 and 14 SNPs associated with cough, dyspnea and phlegm respectively at a *p <* 10^−4^ significance level. In the meta-analysis of two independent replication cohorts, one association was observed between cough and rs16918212 located on chromosome 12 in intron of *A2MP1*, and no associations with dyspnea and phlegm were replicated.

The odds ratio for this SNP indicates that carriers of the A allele have a lower risk to cough than subjects with the wild type genotype. This SNP is located in an intron of *A2MP1* (alpha-2-macroglobulin pseudogene 1). *A2MP1* has been associated with Alzheimer’s disease [25]. Pseudogenes are genomic DNA sequences similar to normal genes but non-functional; they have lost their gene expression in the cell or their ability to code protein [26]. Some pseudogenes can be functional when they are transcribed. Increasing evidence suggests that pseudogenes may have important physiological functions [26].

A major strength of this study the fact this is the first GWA study trying to identify genetic susceptibility loci for cough, dyspnea and phlegm, which included 2 verification samples: one using the same methodology (LifeLines II) and one using similar methodology (Vlagtwedde-Vlaardingen) as the discovery sample (LifeLines I). The respiratory symptoms that we studied were defined based on the standardized questionnaire of the ECRHS.

A GWA study has the advantage of being hypothesis-free. This means that it has the potential of finding new genes underlying disease phenotypes [27]. However, GWA studies also have some disadvantages such as the need of a large study sample, the need for replication, the inability to address causation, and the inability to investigate rare genetic variants [27].

A limitation of our study might be the fact that we used a liberal *p-*value threshold (*p <* 10^−4^) for identification of SNPs in the identification cohort to keep the risk of not detecting a true association between genetic markers and respiratory symptoms low. However, when we assessed these associations in the replication cohorts, the total number of significant associations in the replication meta-analysis is less than expected by chance (i.e. 1 out of the 50 SNPs analyzed for replication (i.e. 2%) had a *p-*value < 0.05 and the same direction of effect as in the identification analysis). In addition, given that rs16918212 and the *A2MP1* gene have not been associated with lung function impairment or respiratory diseases we think the association is likely not a true finding. We therefore conclude that there was no convincing association between genetic markers and respiratory symptoms in this study.

The lack of finding a plausible significant association between SNPs and respiratory symptoms can possibly be explained by the fact that a respiratory symptom can be caused by different environmental exposures or can be a presentation of different underlying diseases with specific genetic or environmental origins. For example, cough, can be triggered by smoking, air pollution and occupational exposures. Susceptibility to these various exposures may be genetically determined and susceptibility loci may differ between exposures. In addition, cough is a common symptom of several chronic respiratory conditions such as asthma, chronic obstructive pulmonary disease (COPD), and lung cancer [28], but cough is also present in non-respiratory conditions such as heart failure [29]. Dyspnea is a common symptom not only in patients with lung and heart diseases, but it is also fairly prevalent among elderly individuals without apparent pre-existing disease [5].

## Conclusion

We did not find a convincing association between genetic markers and the presence of respiratory symptoms cough, dyspnea and phlegm. This lack of association between genetic variants and respiratory symptoms may possibly be due to the fact that we did not take the effect of environmental exposures that give rise to respiratory symptoms into account. Therefore, the next logical step will be performing a genome-wide interaction (GWI) study to identify genetic loci for respiratory symptoms in interaction with known harmful environmental exposures.

## Additional file

Additional file 1: Manhattan plots and additional analyses on chronic cough and phlegm. (DOCX 584 kb)

## Abbreviations

*SNP*: Single nucleotide polymorphism *GWAS*, Genome wide association study
