# Supplementary material for: No convincing association between genetic markers and respiratory symptoms: results of a GWA study
Source: Respir Res. 2017 Jan 10;18:11. doi: 10.1186/s12931-016-0495-4 (PMC5223330; doi:10.1186/s12931-016-0495-4)
Supplement: Additional file 1: — Manhattan plots and additional analyses on chronic cough and phlegm. (DOCX 584 kb) [file 12931_2016_495_MOESM1_ESM.docx]

**Supplemental material**

**No convincing association between genetic markers and respiratory symptoms: results of a GWA study**

**Xiang Zeng, Judith M. Vonk, Kim de Jong, Xijin Xu , Xia Huo, H. Marike Boezen**

**Table S1.** Top SNPs (n=16) associated with cough for at least 3 months per year in the GWA study (all P < 1.0×10^-4^)

| SNPs | Chr | Gene | A1 | MAF | Identification  LifeLines I  674 cases/7095 controls | | Replication | | | | Meta-analysis  Replication | |
| --- | --- | --- | --- | --- | --- | --- | --- | --- | --- | --- | --- | --- |
|  |  |  |  |  |  |  | LifeLines II  406 cases/4657 controls | | Vlagtwedde-Vlaardingen  148 cases/1373 controls | |  |  |
|  |  |  |  |  | OR | P | OR | P | OR | P | OR | P |
| rs1505353 | 2 | ERBB4 | A | 0.37 | 1.286 | 1.53E-05 | 0.989 | 0.890 | 1.157 | 0.245 | 1.033 | 0.623 |
| rs10982541 | 9 | TNC | T | 0.06 | 1.579 | 2.26E-05 | 1.145 | 0.362 | 0.960 | 0.883 | 1.101 | 0.462 |
| rs17308541 | 14 | 3.9kb 5' of EDDM3A | G | 0.12 | 1.399 | 3.21E-05 | 0.842 | 0.153 | 0.586 | 0.0179 | 0.777 | 0.018 |
| rs3803815 | 17 | RP11-74H8.1 | T | 0.07 | 1.519 | 4.05E-05 | 1.113 | 0.476 | 0.952 | 0.837 | 1.065 | 0.620 |
| rs2858016 | 16 | NA | T | 0.22 | 1.309 | 4.54E-05 | 1.028 | 0.760 | NA | NA | NA | NA |
| rs4900348 | 14 | VRK1 | A | 0.41 | 0.781 | 5.03E-05 | 0.941 | 0.422 | 1.354 | 0.017 | 1.035 | 0.601 |
| rs7304994 | 12 | A2ML1 | G | 0.20 | 1.319 | 5.52E-05 | 0.990 | 0.914 | 0.991 | 0.955 | 0.990 | 0.903 |
| rs4678512 | 3 | 26kb 5' of FBXL2 | T | 0.11 | 1.412 | 6.98E-05 | 0.815 | 0.110 | 0.758 | 0.207 | 0.801 | 0.044 |
| rs1944663 | 11 | RP11-179A16.1 | C | 0.06 | 1.51 | 7.03E-05 | 1.009 | 0.949 | 0.812 | 0.415 | 0.955 | 0.721 |
| rs2383971 | 8 | 17kb 5' of RP11-706J10.1 | T | 0.42 | 1.259 | 7.08E-05 | 1.045 | 0.560 | 1.135 | 0.318 | 1.068 | 0.313 |
| rs2305351 | 4 | EPHA5 | T | 0.14 | 1.354 | 7.27E-05 | 1.001 | 0.989 | 0.790 | 0.204 | 0.944 | 0.532 |
| rs9944047 | 14 | RP11-433J8.2 | C | 0.49 | 1.259 | 7.38E-05 | 1.022 | 0.766 | 0.952 | 0.690 | 1.003 | 0.964 |
| rs4756804 | 11 | 39kb 3' of INSC | C | 0.04 | 1.689 | 7.92E-05 | 0.832 | 0.373 | 1.261 | 0.465 | 0.941 | 0.727 |
| rs3758054 | 8 | 1.5kb 5' of HNF4G | G | 0.29 | 1.275 | 8.11E-05 | 0.984 | 0.844 | 1.187 | 0.201 | 1.034 | 0.626 |
| rs6755027 | 2 | ERBB4 | A | 0.46 | 1.253 | 9.04E-05 | 1.004 | 0.954 | 0.898 | 0.393 | 0.975 | 0.694 |
| rs263315 | 2 | 75kb 5' of GALNT5 | G | 0.12 | 1.379 | 9.52E-05 | 0.817 | 0.098 | 0.978 | 0.912 | 0.858 | 0.141 |

**Table S2.** Top SNPs (n=28) associated with phlegm for at least 3 months per year in the GWA study (all P < 1.0×10^-4^)

| SNPs | Chr | Gene | A1 | MAF | Identification  LifeLines I  546 cases/7254 controls | | Replication | | | | Meta-analysis  Replication | |
| --- | --- | --- | --- | --- | --- | --- | --- | --- | --- | --- | --- | --- |
|  |  |  |  |  |  |  | LifeLines II  356 cases/4726 controls | | Vlagtwedde-Vlaardingen  114 cases/1407 controls | |  |  |
|  |  |  |  |  | OR | P | OR | P | OR | P | OR | P |
| rs4818199 | 21 | 79kb 3' of LINC00323 | T | 0.45 | 0.717 | 4.84E-07 | 0.930 | 0.359 | 0.993 | 0.963 | 0.945 | 0.409 |
| rs16856619 | 3 | 647kb 5' of RP11-88H10.2 | A | 0.11 | 1.47 | 1.65E-05 | 1.237 | 0.073 | 0.789 | 0.301 | 1.125 | 0.264 |
| rs6755276 | 2 | AC009499.1 | C | 0.24 | 0.708 | 1.75E-05 | 0.973 | 0.761 | 1.130 | 0.447 | 1.009 | 0.910 |
| rs6808937 | 3 | 32kb 5' of RP11-644C3.1 | A | 0.08 | 1.561 | 1.82E-05 | 1.071 | 0.637 | 1.152 | 0.564 | 1.092 | 0.484 |
| rs7292 | 22 | MB | A | 0.46 | 0.762 | 2.19E-05 | 0.787 | 0.003 | 1.111 | 0.457 | 0.855 | 0.024 |
| rs2839467 | 21 | UMODL1 | A | 0.24 | 1.349 | 2.30E-05 | 1.047 | 0.613 | 0.987 | 0.937 | 1.033 | 0.685 |
| rs9826769 | 3 | 695kb 5' of RP11-88H10.2 | A | 0.11 | 1.459 | 2.77E-05 | 1.241 | 0.067 | 0.788 | 0.297 | 1.128 | 0.250 |
| rs3934600 | 9 | FAM189A2 | A | 0.16 | 1.390 | 2.91E-05 | 0.945 | 0.608 | 1.039 | 0.841 | 0.968 | 0.731 |
| rs933741 | 12 | 11kb 3' of RP11-385N17.1 | G | 0.16 | 0.659 | 3.59E-05 | 1.061 | 0.574 | 1.164 | 0.416 | 1.085 | 0.374 |
| rs1929896 | 9 | 3.5kb 3' of FAM189A2 | T | 0.16 | 1.384 | 3.71E-05 | 0.958 | 0.696 | NA | NA | NA | NA |
| rs9879244 | 3 | 29kb 5' of RP11-644C3.1 | A | 0.07 | 1.563 | 4.22E-05 | 1.072 | 0.654 | 1.109 | 0.685 | 1.082 | 0.553 |
| rs11747502 | 5 | 48kb 3' of CTD-2203K17.1 | T | 0.20 | 0.701 | 5.23E-05 | 0.894 | 0.264 | 1.114 | 0.506 | 0.950 | 0.551 |
| rs10933532 | 2 | PASK | T | 0.07 | 1.537 | 5.36E-05 | 0.857 | 0.322 | 0.770 | 0.344 | 0.835 | 0.185 |
| rs7605293 | 2 | 47kb 3' of AC013727.1 | G | 0.14 | 1.411 | 5.55E-05 | 1.016 | 0.887 | 1.092 | 0.638 | 1.036 | 0.717 |
| rs6932636 | 6 | TRERF1 | C | 0.15 | 0.666 | 5.73E-05 | 0.918 | 0.433 | 0.676 | 0.074 | 0.864 | 0.133 |
| rs9467046 | 6 | 6.1kb 5' of NRSN1 | G | 0.50 | 1.291 | 5.81E-05 | 1.083 | 0.305 | 1.047 | 0.732 | 1.074 | 0.291 |
| rs7477796 | 10 | NRG3 | T | 0.26 | 0.734 | 6.34E-05 | 1.013 | 0.884 | 1.068 | 0.666 | 1.027 | 0.732 |
| rs912465 | 9 | 39kb 3' of HSD17B3 | A | 0.30 | 1.306 | 6.63E-05 | 1.026 | 0.766 | 1.144 | 0.376 | 1.053 | 0.486 |
| rs12956032 | 18 | 6kb 5' of RN5S461 | T | 0.11 | 1.430 | 6.70E-05 | 1.038 | 0.771 | 0.722 | 0.208 | 0.966 | 0.766 |
| rs11899268 | 2 | 137kb 3' of AC104076.3 | G | 0.16 | 0.678 | 6.95E-05 | 1.099 | 0.366 | 0.776 | 0.223 | 1.024 | 0.797 |

**Table S2-continued.** Top SNPs (n=28) associated with phlegm for more than 3 months in the GWA study (all P < 1.0×10^-4^)

| SNPs | Chr | Gene | A1 | MAF | Identification  LifeLines I  546 cases/7254 controls | | Replication | | | | Meta-analysis  Replication | |
| --- | --- | --- | --- | --- | --- | --- | --- | --- | --- | --- | --- | --- |
|  |  |  |  |  |  |  | LifeLines II  356 cases/4726 controls | | Vlagtwedde-Vlaardingen  114 cases/1407 controls | |  |  |
|  |  |  |  |  | OR | P | OR | P | OR | P | OR | P |
| rs1368650 | 7 | ABCA13 | A | 0.28 | 1.307 | 7.24E-05 | 1.028 | 0.748 | 0.959 | 0.785 | 1.011 | 0.883 |
| rs2714392 | 18 | 109kb 5' of RP11-739N10.1 | C | 0.09 | 0.597 | 7.42E-05 | 0.924 | 0.571 | 1.676 | 0.016 | 1.104 | 0.399 |
| rs455732 | 6 | REV3L | C | 0.43 | 0.770 | 8.22E-05 | 1.027 | 0.732 | 1.070 | 0.628 | 1.037 | 0.594 |
| rs2071020 | 11 | AMPD3 | T | 0.46 | 1.283 | 8.36E-05 | 0.993 | 0.932 | 1.173 | 0.264 | 1.033 | 0.644 |
| rs10418296 | 19 | ZNF765 | T | 0.16 | 1.363 | 8.52E-05 | 0.990 | 0.922 | 1.061 | 0.742 | 1.008 | 0.934 |
| rs11203184 | 21 | C21orf128 | C | 0.30 | 1.302 | 9.10E-05 | 0.993 | 0.935 | 0.841 | 0.272 | 0.957 | 0.553 |
| rs7938718 | 11 | 1.6kb 3' of RP11-713P17.5 | A | 0.19 | 1.344 | 9.38E-05 | 1.017 | 0.870 | 0.895 | 0.567 | 0.990 | 0.909 |
| rs2909333 | 8 | IDO2 | T | 0.40 | 1.281 | 9.50E-05 | 0.925 | 0.327 | 0.945 | 0.694 | 0.929 | 0.294 |


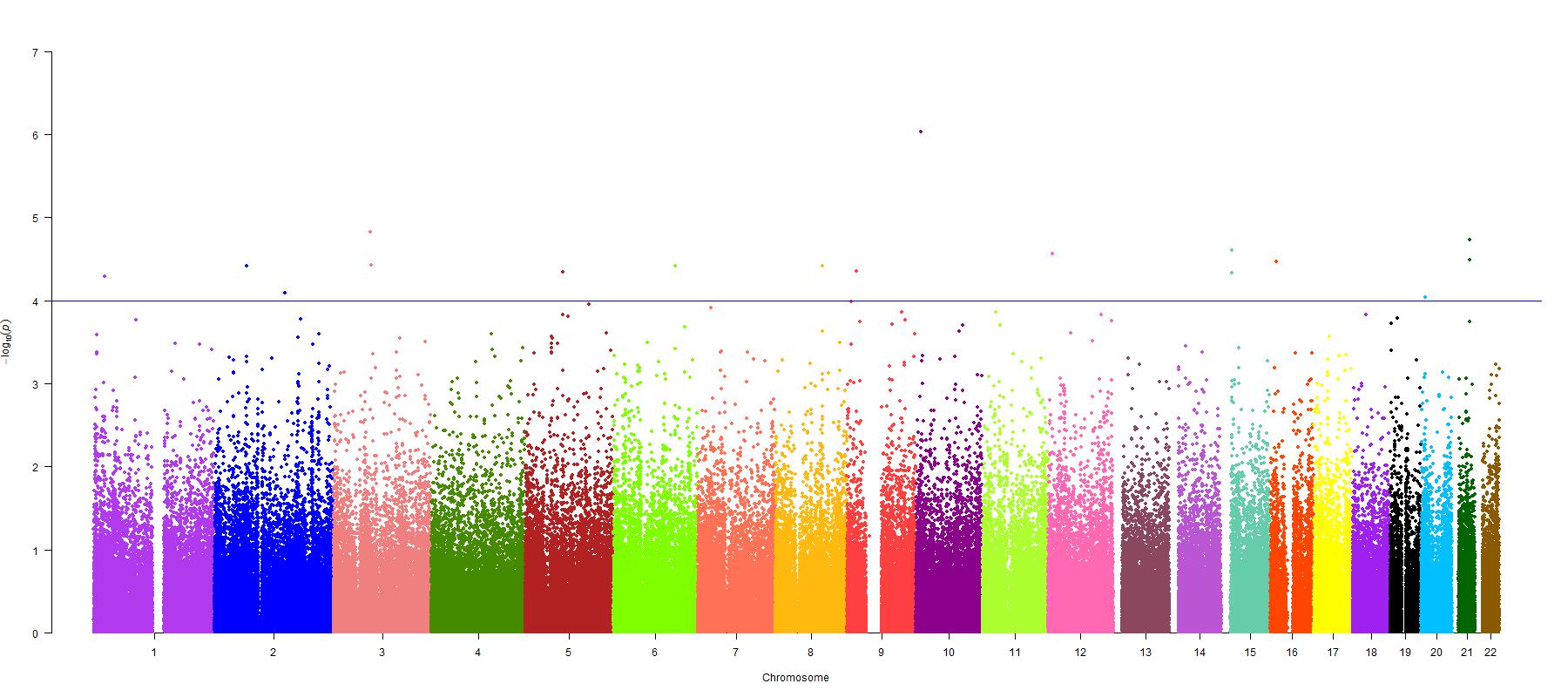


Figure S1. Graphical summary (Manhattan plot) presenting *P*-values for the association between SNPs and cough. The y-axis represents -log10 *P* (logistic regression analysis) from 227,981 SNPs in 7,996 subjects, corrected for age, gender, and current smoking as covariates; the x-axis indicates the SNP’s physical position on successive chromosomes. The Blue line indicates a p value < 10^-4^.


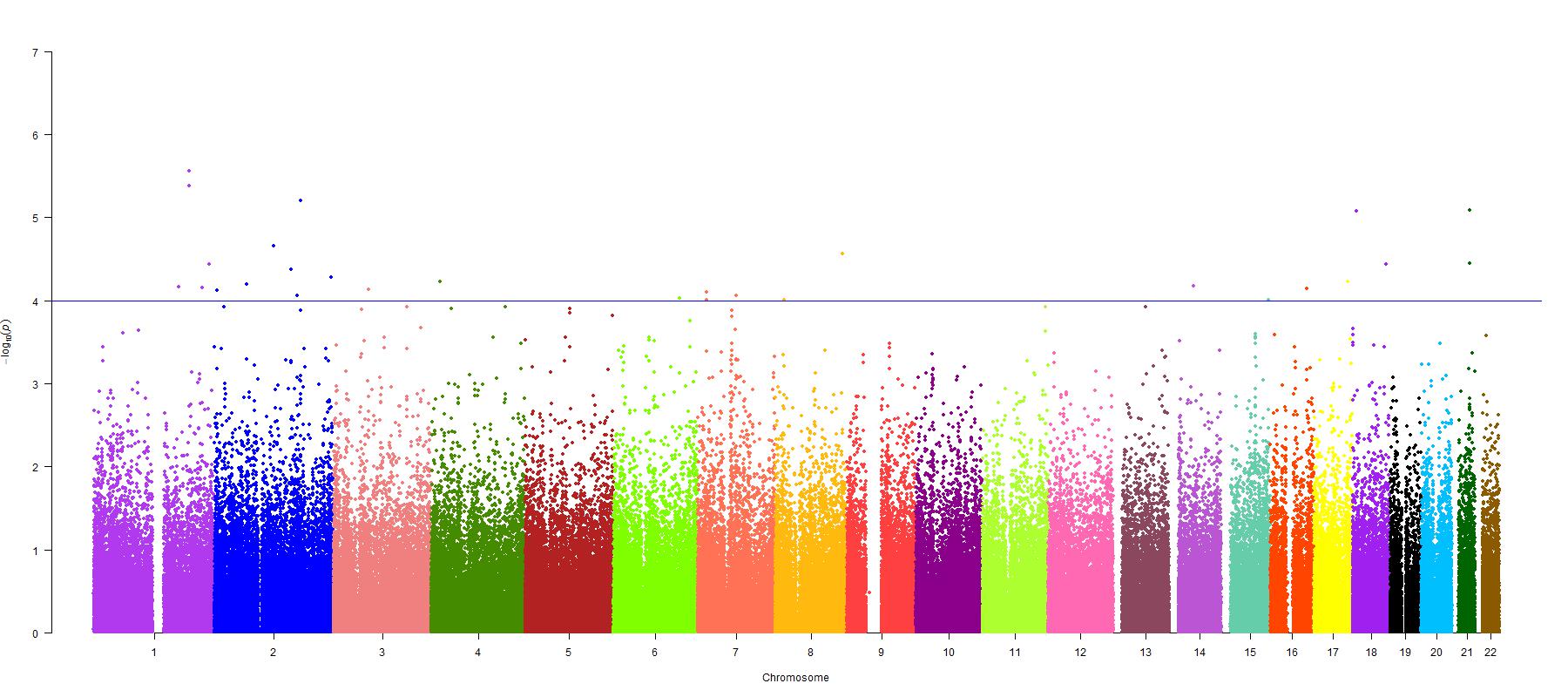


Figure S2. Graphical summary (Manhattan plot) presenting *P*-values for the association between SNPs with dyspnea. The y-axis represents -log10 *P* (logistic regression analysis) from 227,981 SNPs in 7,996 subjects, corrected for age, gender, and current smoking as covariates; the x-axis indicates the SNP’s physical position on successive chromosomes. The Blue line indicates p value < 10^-4^.


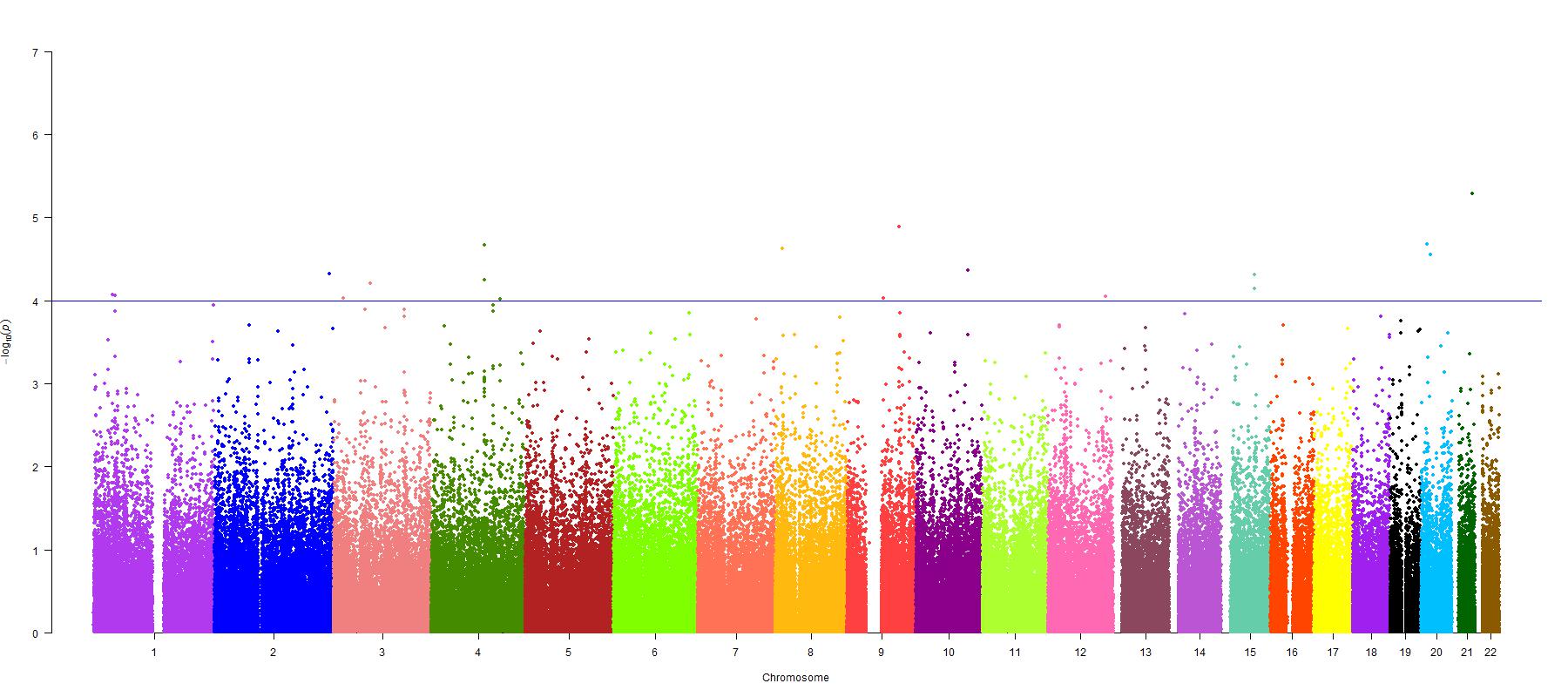


Figure S3. Graphical summary (Manhattan plot) presenting *P*-values for the association between SNPs and phlegm. The y-axis represents -log10 *P* (logistic regression analysis) from 227,981 SNPs in 7,996 subjects, corrected for age, gender, and current smoking as covariates; the x-axis indicates the SNP’s physical position on successive chromosomes. The Blue line indicates a p value < 10^-4^.
